# Characterization of the Difference between Day and Night Temperatures on the Growth, Photosynthesis, and Metabolite Accumulation of Tea Seedlings

**DOI:** 10.3390/ijms24076718

**Published:** 2023-04-04

**Authors:** Xiaoqin Tan, Huili Li, Zhongyue Zhang, Yanjuan Yang, Zhen Jin, Wei Chen, Dandan Tang, Chaoling Wei, Qian Tang

**Affiliations:** 1 College of Horticulture, Sichuan Agricultural University, Chengdu 611130, China; 2 Tea Refining and Innovation Key Laboratory of Sichuan Province, Sichuan Agricultural University, Chengdu 611130, China; 3 The State Key Laboratory of Tea Plant Biology and Utilization, Anhui Agricultural University, Hefei 230036, China

**Keywords:** diurnal temperature range, morphology, physiology, biochemical composition, transcriptome, metabolism

## Abstract

Currently, the effects of the differences between day and night temperatures (DIFs) on tea plant are poorly understood. In order to investigate the influence of DIFs on the growth, photosynthesis, and metabolite accumulation of tea plants, the plants were cultivated under 5 °C (25/20 °C, light/dark), 10 °C (25/15 °C, light/dark), and 15 °C (25/10 °C, light/dark). The results showed that the growth rate of the new shoots decreased with an increase in the DIFs. There was a downward trend in the photosynthesis among the treatments, as evidenced by the lowest net photosynthetic rate and total chlorophyll at a DIF of 15 °C. In addition, the DIFs significantly affected the primary and secondary metabolites. In particular, the 10 °C DIF treatment contained the lowest levels of soluble sugars, tea polyphenols, and catechins but was abundant in caffeine and amino acids, along with high expression levels of theanine synthetase (*TS3*) and glutamate synthase (*GOGAT*). Furthermore, the transcriptome data revealed that the differentially expressed genes were enriched in valine, leucine, and isoleucine degradation, flavone/flavonol biosyntheses, flavonoid biosynthesis, etc. Therefore, we concluded that a DIF of 10 °C was suitable for the protected cultivation of tea plants in terms of the growth and the quality of a favorable flavor of tea, which provided a scientific basis for the protected cultivation of tea seedlings.

## 1. Introduction

The tea plant (*Camellia sinensis* (L.) *O. Kuntze*) is considered to be one of the most important perennial woody plants in China, with a long cultivation history of more than 3000 years [1,2]. Mostly, the tea shoots have been harvested for processing as a beverage, and it has become one of the world’s most popular drinks with an attractive aroma, taste, and health value [3,4,5]. Because tea plants prefer warm, moist, and acidic soil, they require suitable light [6], temperature [7], humidity [8], and soil pH [9] for growth. Among these, temperature is a determining factor in the geographical distribution of the tea plant and restricts its growth. It is well known that there is a large difference in climate between the north and south regions of China [10], which has presented a great challenge for the introduction of tea varieties from the south to the north. Moreover, it could be the main reason why the tea cultivation area in the country cannot fully achieve the coverage of fine tea varieties. In order to break through the natural growth environment barrier, most of the modern agricultural techniques are widely applied in crop cultivation mainly through the control of the light, temperature, water, and soil, which not only shorten the growth cycle but also improve the quality [11,12,13]. The protected cultivation of tea has recently been developed, especially in northern China. Hence, it is necessary to explore suitable temperatures for tea cultivation, so southern tea varieties can also be planted in the north.

The annual average temperature has increased worldwide, year over year, over the last few decades. Temperature is a significant environmental factor for the growth of tea plants [14]. However, recent research has reported that frequent and intense extreme temperatures have resulted in significant economic losses in the tea industry in tea-planting countries [15,16,17]. Therefore, previous studies have mostly focused on growth [18,19], the resilience strategies [20,21], and the regulatory mechanisms of tea plants under temperature stress [22,23]. Temperature stress causes phenotypic, physiological, and biochemical changes in tea plants. Moreover, the diurnal temperature range (DTR) and the difference between day and night temperatures (DIF) also play a critical role in plant growth, and their effects have been well documented in a variety of species, including the finger millet [24], maize [25], rice [26], and wheat [27]. It appears that plants exhibit a number of negative effects in their traits, physiological processes, biochemical components, and productivity when they experience diurnal temperature differences that are higher or lower than optimal. For example, early studies showed that a suboptimal temperature difference severely restricted the growth of shoots and roots [28,29,30], affected the ultrastructure of the chloroplast and the photosynthesis of leaves [31], and led to a yield loss in crops [32]. Furthermore, the DIF was correlated with the carbohydrate translocation in grape leaves, thus affecting the photosynthetic efficiency the following day [33]. Another study conducted by Sunoj [34] on *Zea mays* L. demonstrated that the total soluble sugars and starch were reduced with the narrowing of the diurnal temperature amplitude (from 18 °C to 2 °C), which led to a low-quality yield. Nevertheless, the research on the effects of the DIF on the growth and the development of tea plants is scarce, and this issue needs additional research. In addition, an appropriate temperature difference between day and night could also provide a scientific basis for the optimization of the protected cultivation of tea.

Based on these studies, we hypothesized that tea plants could also be regulated by the DIF. The aim of the present study was to investigate the characteristics of the growth, the photosynthesis, the metabolite accumulation, and the molecular regulatory mechanisms of tea plants (Chuancha No. 2) subjected to three DIFs (day/night, 25/20 °C, 25/15 °C, and 25/10 °C). Furthermore, the results of this study provided new insights into the differences in the metabolisms of tea plants in different DIFs, and a theoretical basis for optimizing the protected cultivation of tea plants in order to produce high-quality tea.

## 2. Results

### 2.1. The Effects of Different DIFs on Tea Plant Growth

**Figure 1:**
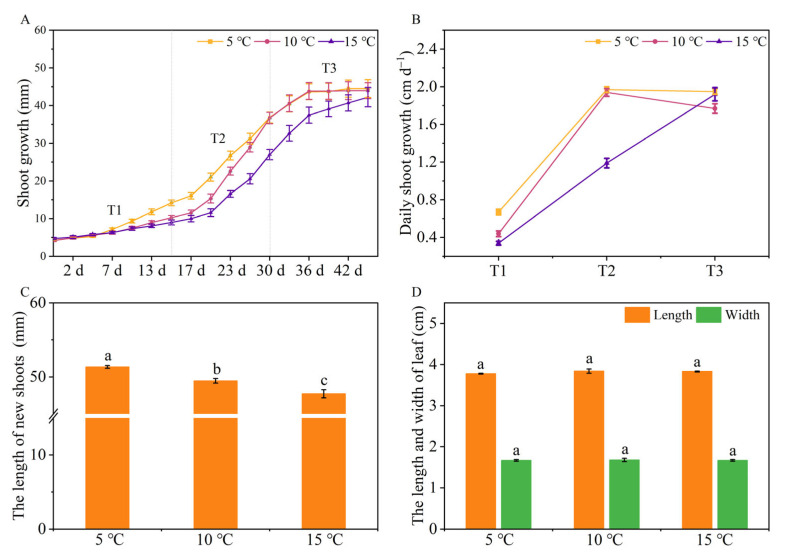
The effects of different DIFs on tea plant growth. (**A**) The length of the new shoots; (**B**) The daily growth rate of the shoots at each stage; (**C**) The length of one bud with three leaves; (**D**) The length and width of the third leaf. Different letters indicate treatments that are significantly different at *p* < 0.05 (*n* = 3).

To explore the growth rate of different treatments, we measured the length of the shoots until the end of the experiment. As shown in Figure 1A,B, the tea seedlings treated with a DIF of 5 °C exhibited the fastest growth rates, followed by the 10 °C and 15 °C treatments. In addition, the growth rates of the tea plants were high at the T2, T2 (16 d~30 d), and T3 (31 d~45 d) stages under the DIFs of 5 °C, 10 °C, and 15 °C, respectively. When the tea seedlings grew to one bud with three leaves, we harvested them and measured their lengths. Figure 1C showed that the length of one bud with three leaves had significantly decreased with the increase in the DIF (*p* < 0.05). However, both the lengths and widths of the third leaf of each treatment had no significant differences (Figure 1D). The results indicated that the increase in the DIF had a negative effect on the growth of the tea seedlings, as evidenced by a rapid growth rate at a DIF of 5 °C and a slow growth rate at a DIF of 15 °C.

### 2.2. The Effects of Different DIFs on the Photosynthetic Indicators, Soluble Sugars, and Protein Contents

**Figure 2:**
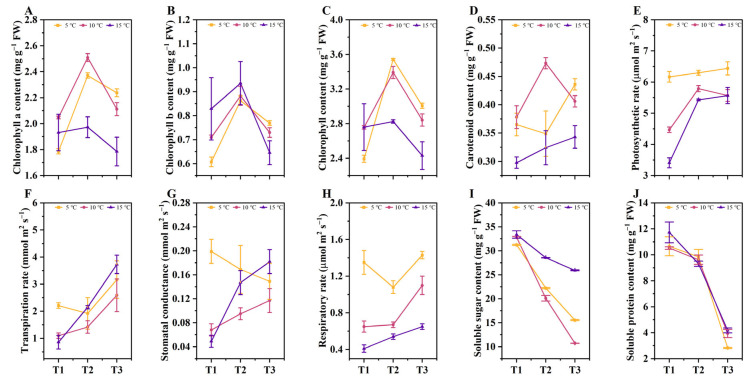
The effects of different DIFs on the photosynthetic indicators, soluble sugars, and proteins. (**A**) Chlorophyll-a content; (**B**) Chlorophyll-b content; (**C**) Total chlorophyll content; (**D**) Carotenoid content; (**E**) Net photosynthetic rate; (**F**) Transpiration rate; (**G**) Stomatal conductance; (**H**) Respiratory rate; (**I**) Soluble sugars; (**J**) Soluble proteins.

The photosynthetic capacities of the mature leaves in all treatments were observed on the 15th, 30th, and 45th day of the treatments. As the tea plants grew, the chlorophyll-a, chlorophyll-b, and total chlorophyll contents in the leaves of each treatment first increased at the T2 (16 d~30 d) stage, and then decreased at the T3 (31 d~45 d) stage (Figure 2A–C). There was a completely different trend in the carotenoid contents in all the treatments (Figure 2D). Specifically, the carotenoid contents in the 5 °C DIF treatment decreased at the T2 stage and then increased at the T3 stage, while the 10 °C DIF treatment showed the opposite trend. In contrast to the 5°C and 10°C DIF treatments, the carotenoid content of the 15 °C DIF treatment increased continuously with the growth of the tea seedlings. In addition, the net photosynthetic rate (Pn), the transpiration rate (Tr), the stomatal conductance (Gs), and the respiration rate (Rr) of the mature leaves of each treatment were determined at three time points. The results showed that increasing the DIF decreased the Pn and Rr, and the order was 5 °C > 10 °C > 15 °C (Figure 2E,H). Interestingly, the Tr of the 15 °C treatment was the lowest in the T1 (1 d~15 d) stage, while it was the highest in both the T2 (16 d~30 d) and T3 (31 d~45 d) stages (Figure 2F). Figure 2G shows the decrease in the Gs in the5 °C DIF treatment during the growth period of the tea seedlings, whereas it increased in the 10 °C and 15 °C DIF treatments. These findings indicated that the increase in the DIF resulted in a decrease in the photosynthesis of the tea seedlings, along with an increase in the respiration.

Additionally, we analyzed the content changes to explore the influence of the DIF on the soluble sugars and proteins of the tea seedlings. The data showed that the contents of soluble sugars in the mature tea leaves gradually decreased with growth development, and this trend was also observed in the soluble proteins (Figure 2I,J). Notably, there was a significant difference (*p* < 0.05) in the soluble sugars among the treatments in the T3 (31 d~45 d) stage, and a maximum content of 25.93 mg g^−1^ FW was reached. Therefore, we speculated that the variations in the DIFs had a greater impact on the soluble sugars than the soluble proteins.

### 2.3. The Effects of Different DIFs on the Contents of Tea Polyphenols, Caffeine, Free Amino Acids, and Catechin Components

**Figure 3:**
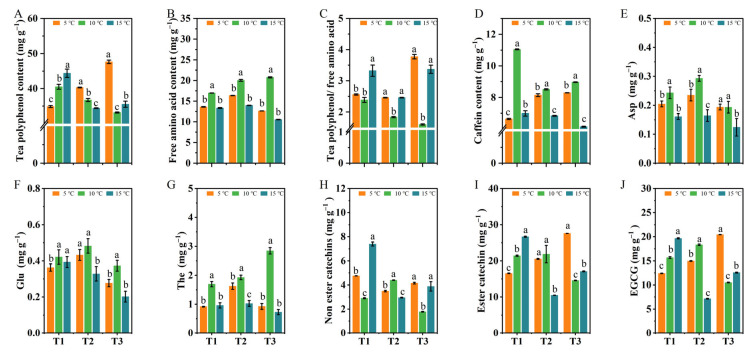
The effects of different DIFs on the contents of biochemical substances, amino acid components, and catechin components. (**A**) Tea polyphenols; (**B**) Free amino acids; (**C**) The ratio of tea polyphenols and free amino acids; (**D**) Caffeine; (**E**) Aspartic acid; (**F**) Glutamate; (**G**) Theanine; (**H**) Non-ester catechin; (**I**) Ester catechin; (**J**) Epigallocatechin gallate. Different letters indicate treatments that are significantly different at *p* < 0.05 (*n* = 3).

In order to comprehensively explore the accumulated characteristics of the metabolites in these treatments, the contents of the quality metabolites, including the tea polyphenols, the free amino acids, and the caffeine of the new shoots, were analyzed. Our results showed that the contents of the tea polyphenols, free amino acids, and caffeine of one bud with three leaves were significantly different (*p* < 0.05) in each of the treatments (Figure 3A,B,D). It was worth noting that the 10 °C DIF treatment contained the highest contents of free amino acids and caffeine at each stage (Figure 3B,D), with the lowest ratio of the tea polyphenols and free amino acids (Figure 3C), suggesting a good tea quality.

In a further analysis, we identified the catechin and amino acid constituents. As a result, a total of 20 amino acids were detected (Figure S1). Three major amino acids, including aspartic acid (Asp), glutamate (Glu), and theanine (The), were the most abundant in the 10 °C DIF treatment, with 0.19~0.29 mg g^−1^ Asp, 0.37~0.48 mg g^−1^ Glu, and 1.70~2.85 mg g^−1^ The (Figure 3E–G, respectively). In total, eight catechins were also obtained in all the treatments, such as gallocatechin (GC), epigallocatechin (EGC), catechin (C), etc. (Figure S2). We found that the non-ester catechins and ester catechins of the tea leaves differed significantly (*p* < 0.05) among the treatments (Figure 3H,I). In particular, epigallocatechin gallate (EGCG) occupied the largest proportion of the total catechins and did not show a regular trend for each treatment, and there were significant differences (Figure 3J). When considered together, these results indicated that the increase in the DIF could affect the contents and constituents of the flavor substances in tea seedlings and contribute significantly to the accumulation of amino acids and caffeine when subjected to a DIF of 10 °C.

### 2.4. Quality Control Analysis of the Transcriptome Data and the Identification of DEGs

**Figure 4:**
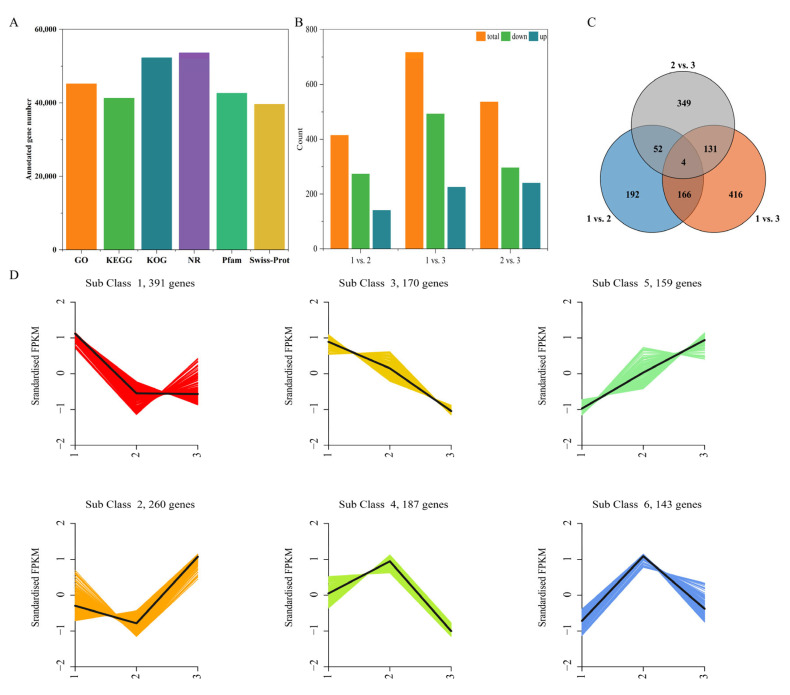
Analysis of the transcriptome data. (**A**) The numbers of annotated genes by GO, KEGG, KOG, NR, Pfam, and Swiss-Prot databases; (**B**) The numbers of differentially expressed genes; (**C**) Venn diagram of the differentially expressed genes among three comparison groups; (**D**) K-means cluster analysis of the differentially expressed genes. Numeral 1 represents the 5 °C DIF treatment; numeral 2 represents the 10 °C DIF treatment; and numeral 3 represents the 15 °C DIF treatment.

To understand the changes in the gene expression of all the treatments in response to different DIFs, the transcriptome profiles of the tea leaves were analyzed using RNA-sequencing. The quality of the transcriptome data of each sample was high, as evidenced by 85.88~86.27% of the genes matching the reference genome, the principal component analysis (PCA), and the square of Pearson’s correlation coefficient (Table S1 and Figure S3). In parallel, the qRT-PCR results validated the reliability of the RNA-sequencing (Figure S4). Using the Gene Ontology (GO), KEGG, KOG, NR, Pfam, and the Swiss-Prot database, a total of 45,174; 41,295; 52,252; 53,658; 42,610; and 39,615 genes were sequentially annotated, respectively (Figure 4A).

The analysis of the DEGs in the three comparison groups, 5 °C vs. 10 °C; 5 °C vs. 15 °C; and 10 °C vs. 15 °C, was performed. In total, 414 DEGs were detected in the 5 °C vs. 10 °C group, including 141 up-regulated and 273 down-regulated genes. There were 717 DEGs identified in the 5 °C vs. 15 °C group, of which 492 were up-regulated and 225 down-regulated, suggesting that it could have been a significantly different metabolic stage. The number of the DEGs in the 10 °C vs. 15 °C group was 536, with 296 up-regulated and 240 down-regulated genes (Figure 4B).

A Venn diagram revealed 4 common DEGs among the 3 comparison groups, named CSS0006640, CSS0009762, CSS0033756, and novel.10871, which probably played a key role in responding to the variations in the DIFs (Figure 4C). Among the 5 °C vs. 10 °C and 5 °C vs. 15 °C groups, the largest number of shared DEGs were screened, totaling 170, followed by the comparative pair of 5 °C vs. 15 °C and 10 °C vs 15 °C. Meanwhile, 416 DEGs were uniquely expressed in the 5 °C vs. 15 °C pair, corresponding to a large decrease in the DIF.

Moreover, we employed a K-means cluster analysis to classify the expression patterns of all the DEGs in each group. Six subclasses were confirmed, which exhibited different expression patterns (Figure 4D). Cluster 1 contained 391 genes that were highly expressed in the of 5 °C DIF group, while there were similar expression levels between the 10 °C and 15 °C DIF groups. In total, 260 genes were included in cluster 2, and the lowest expression level presented in the 10 °C DIF group, with the highest in the 15 °C DIF group. The gene expression level of cluster 3 gradually decreased, at 170, while an opposite trend was presented in cluster 5. In addition, clusters 4 and 6 shared a similar expression pattern. Overall, increasing the DIF could alter the gene expression patterns and possibly influence the metabolites of the tea leaves.

### 2.5. Analysis of the Differentially Expressed Transcription Factors (TFs) and the Function of the DEGs

**Figure 5:**
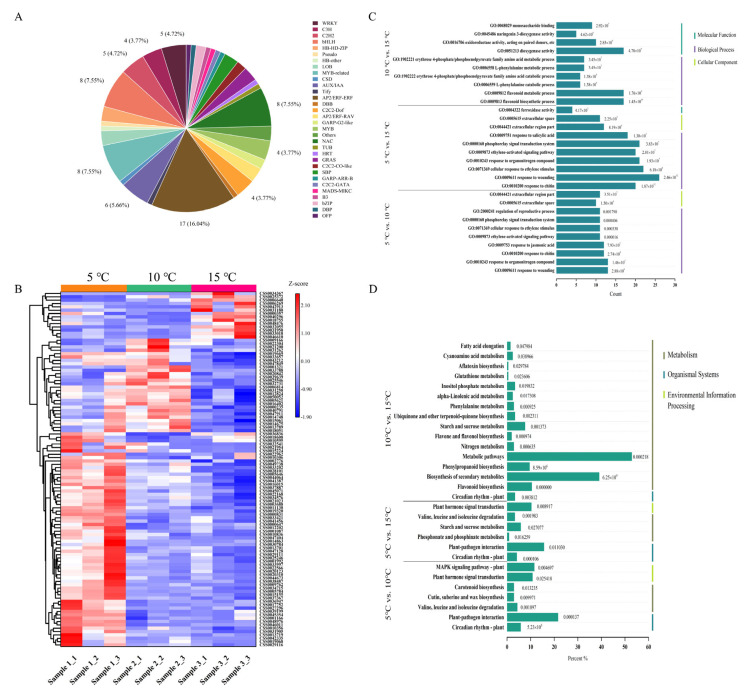
Annotation of the differentially expressed genes. (**A**) The classification of the differentially expressed transcription factors; (**B**) The clustering heat map of the differentially expressed transcription factors; (**C**) GO enrichment analysis for the DEGs between the comparison groups; (**D**) KEGG enrichment analysis for the DEGs between comparison groups. Numeral 1 represents the 5 °C DIF treatment; numeral 2 represents the 10 °C DIF treatment; and numeral 3 represents the 15 °C DIF treatment.

Transcription factors (TFs) are essential regulators of the plant metabolism. A total of 106 differentially expressed genes were predicted as TFs and grouped into 32 families, including WRKY, bHLH, MYB, NAC, AUX/IAA, etc. (Figure 5A). The AP2/ERF-ERF, bHLH, MYB-related, and NAC families contained the largest numbers of TFs, at 17, 8, 8, and 8, respectively. Regarding the gene expression levels of the TF family members, all were clustered into three categories (Figure 5B). Among them, 64 TFs were significantly upregulated in the 5 °C DIF group, followed by 28 TFs in the 10 °C DIF group, and 14 TFs in the 15 °C DIF group. Therefore, with the increase in the DIF, the number of highly expressed TFs in the tea leaves gradually decreased, suggesting that the slower metabolic process of the tea plants was probably induced by the lower night temperature.

To analyze the biological functions of the DEGs obtained in this study, a GO analysis was performed. Based on the GO annotations, Figure 5C shows the first 10 significantly enriched terms (*p* < 0.05) among the three comparison pairs, most of which were in the biological processes, followed by the molecular functions, and the cellular components. In the 5 °C vs. 10 °C group, similar numbers of DEGs were involved in the response to wounding (GO: 0009611), the response to organonitrogen compounds (GO: 0010243), the response to jasmonic acid (GO: 0009753), etc. In addition, the GO terms such as response to wounding (GO: 0009611), the cellular response to ethylene stimulus (GO: 0071369), and the phospho-relay signal transduction system (GO: 0000160) showed prominent enrichment in the 5 °C vs. 15 °C group. Notably, the largest number of DEGs was significantly enriched in the flavonoid biosynthetic process (GO: 0009813), the flavonoid metabolic process (GO: 0009812), and the dioxygenase activity (GO: 0051213) between the 10 °C and 15 °C groups, suggesting a strong influence on the regulation of the secondary metabolites of the tea leaves affected by the DIF.

Furthermore, the KEGG pathway analysis was performed to investigate the major metabolic pathways of the tea plants in different treatments. A total of 29 pathways were significantly enriched and classified into 3 categories, including metabolism, environmental information processing, and organismal systems (Figure 5D). In detail, 7, 6, and 16 pathways of the DEGs were significantly enriched in the 5 °C vs. 10 °C, 5 °C vs. 15 °C, and 10 °C vs. 15 °C (*p* < 0.05), respectively. Between the 5 °C vs. 10 °C and the 5 °C vs. 15 °C groups, the DEGs were enriched in common pathways, such as the plant hormone signal transduction, as well as the valine, leucine, and isoleucine degradation. Nevertheless, the starch and sucrose metabolism as well as the circadian rhythm pathways were commonly enriched in the both 5 °C vs. 15 °C and 10 °C vs. 15 °C groups. Significantly, the metabolic pathway as well as the flavone and flavonol biosyntheses, the flavonoid biosynthesis, and the starch and sucrose metabolism, which are closely related to tea flavor quality, were all annotated in the DIFs of the 10 °C vs. 15 °C group. Hence, the KEGG analysis results revealed that the DIF could affect the metabolisms of the flavonoids and their derivatives, which was consistent with the GO enrichment analysis.

### 2.6. Regulation of the DEGs in the Plant Hormone Signal Transduction

**Figure 6:**
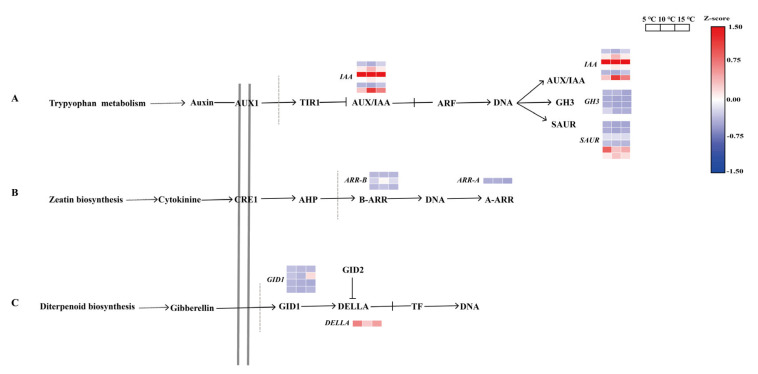
The expression patterns of genes in the plant hormone signal transduction under different DIF treatments.

According to the DEG annotation analysis, the plant hormone signal transduction responded to the variations in the DIF. Furthermore, the gene expression profiles in this pathway were analyzed. In total, 46 DEGs were obtained from the transcriptome data that were closely related to the biosynthesis to the plant hormones, including auxin, gibberellin, cytokinin, jasmonic acid, abscisic acid, ethylene, salicylic acid, and brassinosteroids (Table S2). For example, we identified six genes (CSS0010306, CSS0019669, CSS0019320, CSS0016015, CSS0000357, and CSS0000235) of the auxin-responsive protein IAA (*IAA*), and four (CSS0001919, CSS0044950, CSS0024964, and CSS0032471) of the auxin-responsive GH3 gene family (*GH3*), all of which belong to the auxin early response protein (Figure 6A). Moreover, most of the 6 genes in the SAUR family protein (*SAUR*) were highly expressed in the 5 °C DIF treatment, while the 4 genes of the 2-component response-regulator ARR-A/B family (*ARR-A/B*) had the lowest expression pattern in the 15 °C DIF treatment (Figure 6B). With the exception of CSS0004492, 3 *GID1* and 1 *DELLA* had the highest expression in the 5 °C DIF treatment (Figure 6C). In addition, eight genes (CSS0010595, CSS0009130, CSS0043212, CSS0047119, CSS0010246, CSS0010469, novel.5176, and CSS0049233) involved in the brassinosteroid biosynthesis were identified, and most of their expressions gradually decreased with the increasing DIF. Surprisingly, a total of 11 (CSS0049233, CSS0009166, CSS0029111, CSS0017736, CSS0039257, CSS0002402, CSS0015224, CSS0012203, CSS0002809, CSS0037023, and CSS0011319) DEGs were involved in jasmonic acid, abscisic acid, ethylene, salicylic acid, and brassinosteroid biosyntheses, as well as in the MAPK signaling pathway (Table S2).

### 2.7. Regulation of the DEGs in the Catechin Biosynthesis Pathway

**Figure 7:**
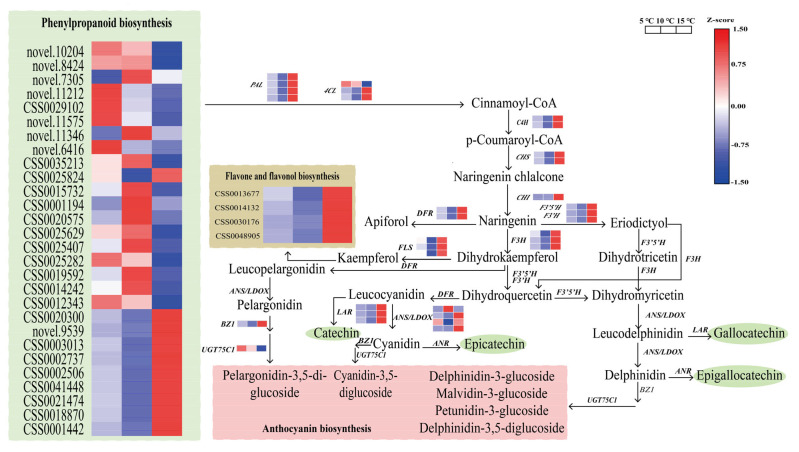
The expression patterns of genes in the flavonoid pathway in the 5 °C, 10 °C, and 15 °C DIF treatments.

To elucidate the molecular mechanism of catechin, we investigated the expression patterns of the DEGs involved in the flavonoid pathways among the three groups (Figure 7). In the flavonoid biosynthesis pathway, 32 DEGs were identified, among which 29 DEGs had the highest expression levels in the 15 °C DIF treatment. In the initial steps of the pathway, 4 DEGs for coding phenylalanine ammonia-lyase (*PAL*), 3 for coding 4-coumarate-coa ligase gene (*4CL*), and 2 for coding cinnamate 4-hydroxylase gene (*C4H*), respectively, were identified. Except for 1 transcript of genes encoding *4CL*, all 8 genes were expressed at the highest level in the 15 °C DIF treatment, with the lowest level in the 10 °C DIF treatment. Next, the DIF affected the expression patterns of *CHS* (CSS0004474 and CSS0007714) and *CHI* (novel.12866), which were significantly upregulated in the 15 °C DIF treatment, as compared to the 5 °C and 10 °C DIF treatments. Flavonoid 3′,5′-hydroxylase (F3′5′H) and flavonoid 3′-monooxygenase (F3′H) catalyzed the biosyntheses of dihydromyricetin and dihydroquercetin, respectively, both of whose genes had the highest expression levels in the 15 °C DIF treatment, with the lowest in the 10 °C DIF treatment. In addition, 3 flavonol synthase genes (*FLS*), including CSS0045924, CSS0007745, and CSS0046529, were expressed in the following ranking order: 15 °C > 5 °C > 10 °C, as well as 2 of the 4-reductase (*DFR*). Leucoanthocyanidin reductase (LAR) was responsible for catalyzing the biosynthesis of catechin, whose gene expression analysis showed that CSS0013831, CSS0028235, and CSS0009063 expressed the lowest levels in the 10 °C DIF treatment, and the abundance of catechin showed a similar trend. We then identified 4 DEGs (CSS0046216, CSS0029211, CSS0010687, and CSS0007481) for encoding LDOX, whose expression levels differed significantly in each treatment. The anthocyanidin reductase (*ANR*) was involved in the biosynthesis of both catechins, but no DEGs were found in this scenario. Additionally, anthocyanidin 3-O-glucosyltransferase (*BZ1*) and *UGT75C1* were also differentially expressed in the present study, with the highest level of BZ1 and the lowest level of anthocyanidin 3-O-glucoside 5-O-glucosyltransferase (*UGT75C1*) in the 15 °C DIF treatment. These results indicated that the differential expression patterns of the genes related to the flavonoids resulted in a low content of catechins in the 10 °C DIF treatment.

### 2.8. Regulation of the DEGs in the Amino Acid Metabolic Pathway

**Figure 8:**
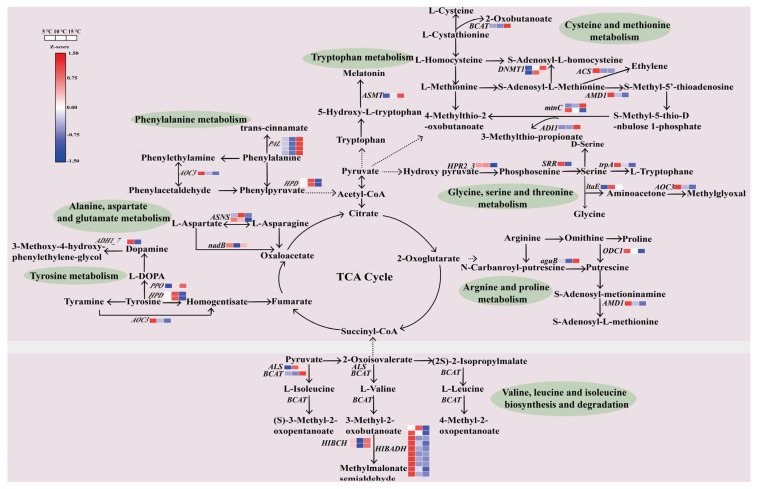
The expression patterns of genes in amino acid metabolism in the 5 °C, 10 °C, and 15 °C DIF treatments.

Based on the KEGG analysis, a total of 11 enriched pathways (including 46 DEGs) were analyzed in order to investigate the patterns of the genes involved in the amino acid metabolic pathways (Table S3). Among them, the valine, leucine, and isoleucine degradation were significantly enriched in 2 compared pairs, the 5 °C vs. 10 °C DIF and the 5 °C vs. 15 °C DIF (Figure 5D). Specially, one branched-chain amino acid aminotransferase (*BCAT*, CSS0023496), two 3-hydroxyisobutyryl-CoA hydrolases (*HIBCH*, CSS0034648 and CSS0038802), and ten 3-hydroxyisobutyrate dehydrogenases (*BIBADH*, CSS0005724, CSS0005847, CSS0013914, CSS0015917, CSS0026745, CSS0031378, CSS0031667, CSS0034869, CSS0039835, and CSS0042958) genes were identified, with the highest expression level of *BCAT* and *HIBCH*, and the lowest level of seven genes of *HIBADH* in the 15 °C DIF treatment. Meanwhile, 1 *ALS* (novel.11593) and 1 *BCAT* (CSS0023496) participated in the biosyntheses of L-Isoleucine, L-Valine, and L-Leucine, which were highly expressed in the 10 °C and 15 °C DIF treatments, respectively. The tea leaves from each treatment had accumulated a large amount of aspartic acid, glutamate, and theanine (Figure 3E–G). In this work, two genes encoding asparagine synthase (*ASNS*) and one gene encoding L-aspartate oxidase (*nadB*) were differentially expressed; these were involved in alanine, aspartate, and glutamate metabolisms. As compared to the other groups, the expression level of *nadB* in the 5 °C DIF treatment was 1.04-fold higher than that in the 10 °C DIF treatment, and it increased by 10.24%, as compared to the 15 °C DIF treatment (Figure 8). Although the DEGs related to the theanine metabolism were not obtained from the transcriptome data, we detected the expression of 5 structural genes mediating theanine biosynthesis, including glutamate synthase (*GOGAT*), glutamine synthetase (*GS*), theanine synthetase (*TS1* and *TS3*), and theanine hydrolase (*ThYD*) (Figure S5). It was found that both *TS3* and *GOGAT* were highly expressed in the 10 °C DIF treatment. The expressions of *GS2* and *TS1* had a similar trend among treatments, with the highest levels in the 5 °C DIF treatment. In contrast, *ThYD* was involved in the decomposition of theanine, which showed an elevated expression with the increase in the DIFs. When considered altogether, these results could indicate an accumulation of theanine in the tea leaves when treated with a DIF of 10 °C.

### 2.9. Regulation of the DEGs in the Caffeine Metabolic Pathway

Caffeine is also one of the flavor substances of tea, not limited to tea polyphenols, catechins, and amino acids. It was found that only one DEG-encoding caffeine synthase involved in caffeine synthesis, novel.165, showed higher expression levels in the 5 °C and 10 °C DIF treatments (Table S4), which was beneficial for the caffeine accumulation.

## 3. Discussion

In the current study, the DIF influenced the tea seedlings’ growth, as evidenced by the reduced daily growth rate and length in one bud with three leaves, with an increase in the DIF. This was supported in a study conducted by Liu et al. [35], who reported that a DIF of 15 °C markedly inhibited the growth of the shoots and stems of both *A. membranaceus* and *C. lanceolata*. In the process of growth and development, plants regulate their metabolisms via phytohormones in order to adapt to their external environment [36,37]. Auxin (IAA) is known to play a key role in cell differentiation, division, and enlargement in the root, stem, and shoot. Gibberellin (GA) plays a role in plant stem elongation. It was found that stem elongation under different DIF treatments was accompanied by different GA and IAA levels, and even the expression patterns of the genes related to GA and IAA syntheses [38]. Therefore, most of the IAA-encoding genes were highly expressed under a DIF of 5 °C, whereas the genes related to GA were expressed at low levels (Figure 6). Similarly, the results were consistent with a study on tomato plants reported by Ohtaka et al. [38]. Based on these results, we speculated that the IAA biosynthesis-related genes might be more dominant in stem elongation than the GA biosynthesis-related genes in this study. However, our hypothesis still needs to be confirmed by further investigation. Earlier studies also reported the role of brassinosteroids (BRs) and cytokinins (CTKs) in regulating cell division and elongation. Moreover, 8 DEGs (*TCH4, BAK1,* and *BRI1*) engaged in brassinosteroid biosynthesis, and 4 DEGs (*ARR-A/B*) involved in cytokinin biosynthesis were in the lowest expression pattern in the 15 °C DIF treatment, which probably resulted in the inhibition of shoot growth in the tea seedlings. Consequently, it was concluded that the difference in growth among different DIFs could be attributed to the regulation of IAA and GA, combined with CTKs and BRs.

The photosynthetic capacity was closely related to plant growth and development and affected by the DIFs [39,40,41]. According to our findings, the DIFs influenced the photosynthetic capacity of the mature leaves of the tea seedlings. For example, with the development of the tea plants, chlorophyll a, chlorophyll b, and the total chlorophyll of each treatment accumulated significantly after 16~30 days, which was conducive to a high net photosynthetic rate until the end of the experiment, especially at a DIF of 5 °C (Figure 2A–C). Meanwhile, the respiration rate decreased with the increase in the DIF, which indicated an increase in the photosynthetic products [42,43], such as the soluble sugars, in the 15 °C DIF treatment (Figure 2I). Despite the reduction in the photosynthesis with the increase in the DIF, the respiration rate declined and consumed less, which could explain the abundance of soluble sugars in the 15 °C DIF treatment.

In this study, a large DIF was not beneficial for the accumulation of the tea polyphenols and catechins, which was different from the results of Jeong et al. [44]. To date, the biosynthetic pathway of catechins has been well-characterized [45,46] and appeared to be regulated by a variety of genes [47]. We found that the catechins accumulated the least in the 10 °C DIF treatment with the change in the DIF, whereas 27 DEGs involved in the early, middle, and late stages of the flavonoid pathway had the lowest expression levels (Figure 7). Therefore, we assumed that the low expressions of these genes caused the low catechin content. A previous study proved that leucoanthocyanidin reductase (LAR) catalyzed the biosyntheses of catechin (C) and gallocatechin (GC) [48]. Here, 3 genes of *LAR* from the 10 °C DIF treatment had the lowest expression levels, which was consistent with the low abundance of C and GC. It was demonstrated that anthocyanidin reductase (*ANR*) cooperated with anthocyanidin synthase (*ANS*) and dihydroflavonol-4-reductase (*DFR*) to participate in the biosyntheses of epicatechin (EC) and epigallocatechin (EGC) [49]. Although *ANR* was not differentially expressed in this study, 2 *DFR* and 1 *ANS* were identified, with the lowest expression levels of all the genes in the 10 °C DIF treatment, suggesting that it was probably related to the lower contents of EC and EGC. Conversely, no DEGs of *SCPL1A* were obtained among the treatments that converted catechins to CG, ECG, EGCG, and GCG [50]. Similarly, the 4 ester catechins also possessed the lowest level in the 10 °C DIF treatment, which could have been due to the insufficient substrates. Interestingly, 29 DEGs were highly expressed in the 15 °C DIF treatment, which was not in line with the catechin content. According to the gene expression patterns of the flavonoid pathway branch, we speculated that the enhancement of the branch metabolism (e.g., flavone and flavone biosyntheses, and anthocyanin biosynthesis) could have led to lower catechins in the 15 °C DIF treatment, as compared to the 5 °C DIF treatment. Since we did not detect all the metabolites in the pathway in this study, this needs to be further investigated.

On the contrary, the 10 °C DIF treatment was beneficial for the amino acid accumulation in the tea seedlings. It has been widely understood that amino acid biosynthesis was associated with nitrogen metabolism [51,52]. The significantly enriched pathway of nitrogen metabolism provided a sufficient nitrogen source for the biosynthesis of nitrogen compounds, where the carbonic anhydrase (CA) and nitrate/nitrite transporter (Nrt) has played indispensable roles in the utilization of carbon dioxide and the transfer of carboxylic acid [53,54]. In particular, the expression of CSS0049176 (*CA*) in the 10 °C DIF treatment was remarkably 3-fold higher than in the other treatments, which may have been critical for synthesizing more amino acids (Table S5). In addition, 10 DEGs of the 3 treatments were enriched in the glycolysis cycle (EMP), the tricarboxylic acid cycle (TCA cycle), and the pyruvate metabolism (Table S6), which acted as a bridge to connect the amino acid components [55,56]. In these pathways, all the TCA-cycle-related DEGs in all the treatments were highly expressed and contributed to the biosyntheses of 20 amino acids in 6 major amino acid families. Notably, the valine, leucine, and isoleucine degradation was significantly enriched, as most genes had the highest expression levels in the 5 °C DIF treatment, which was consistent with the lower contents of the 3 amino acids. As a result, this indicated that a lower temperature difference promoted the decomposition of valine, leucine, and isoleucine. An enriched pathway, related to alanine, aspartate, and glutamate metabolism, affected the production of the amino acids in the glutamate family, aspartate family, and alanine family. In these families, aspartic acid, glutamate, and theanine accounted for a large amount in the tea plants. We found that 1 *nadB* and 2 *ASNS* had the lowest expression levels in the 10 °C and 15 °C DIF treatments, respectively, which could have been the reason for the differences in the contents of amino acids (e.g., alanine, aspartic acid, and glutamate) (Figure 8). Furthermore, glutamate and ethylamine together participate in the theanine synthesis through the catalysis of theanine synthase (TS) [57,58]. Unfortunately, there were no differentially expressed genes in the theanine synthesis pathway by transcriptome analysis, and a similar result was also found by Yue et al. [49]. This could have been due to the main synthetic site of theanine being the root, rather than the new shoot that was used in the study [59]. A recent study on the mechanism of theanine biosynthesis showed that tea shoots also had the ability to produce theanine [60]. Moreover, the results of an additional RT-PCR experiment could perhaps explain the highest accumulation of theanine in the 10 °C DIF treatment. In general, an appropriate temperature difference between day and night, especially at 10 °C, promoted amino acid accumulation and reduced degradation via different gene expression patterns.

Caffeine plays an important role in the formation of tea flavor. In the present study, tea leaves from the 10 °C DIF treatment contained more abundant caffeine than those in the 5 °C and 15 °C DIF groups. It is well known that the caffeine synthase gene *TCS* has been reported to convert theobromine to caffeine [61], while herein, only 1 DEG, novel.165, encoding caffeine synthase was upregulated in the 5 °C and 10 °C DIF treatments, but no other reported copies. The gene expression pattern demonstrated that novel.165 could be a dominant gene for caffeine synthesis under different temperature treatments; its underlying regulatory mechanism needs to be further investigated. In addition, the transcription factors, such as MYB, WRKY, bHLH, NAC, etc., were identified in the experiment, which might regulate caffeine, catechin, and theanine synthesis metabolism under different day and night temperatures, and this was supported by previous studies [62,63,64].

## 4. Materials and Methods

### 4.1. Plant Materials and Treatments

In this study, 1-year-old tea seedlings (*Camellia sinensis*, “Chuancha No.2”) were obtained from the Yizhichun Tea Company, Leshan, Sichuan Province (E 103°53′16.3″, N 28°59′24.5″). A total of 270 plants of uniform size and growth were transplanted into plastic pots (length/width/height = 44 cm:20 cm:25 cm) and divided into 3 treatments. Each treatment contained 3 replicates, totaling 90 seedlings. All plants were cultivated in a growth chamber (Shanghai Sanfa Technology Co., Ltd., Shanghai, China) under different temperatures: 5 °C (25/20 °C, light/dark), 10 °C (25/15 °C, light/dark), and 15 °C (25/10 °C, light/dark). Then, 270 tea seedlings were maintained during the same photoperiod of 12/12h (light/dark) at a light intensity of 30,000 lx and relative air humidity of approximately 80%. The experiment lasted 45 days and was divided into 3 stages (0~15 d, T1; 16~30 d, T2; and 31~45 d, T3). After 15, 30, and 45 days of treatments, the mature leaves, along with 1 bud with 3 leaves, were harvested for analysis.

### 4.2. Measurement of Phenotypic Characteristic of Tea Seedlings

A total of 25 buds of similar size were randomly selected and marked. The length of the buds was recorded every 2~3 days, until the end of the experiment. When one bud with three leaves developed, they were weighed, and the length and width of the third leaf under the bud were measured.

### 4.3. Analysis of Physiological Indicators

#### 4.3.1. Determination of Photosynthetic Parameters and Dark Respiration Rate of Mature Leaves

Photosynthetic indicators and dark respiration rates of mature leaves were measured with a portable photosynthesis meter (Li-Cor 6400, Lincoln, NE, USA) at a light intensity of 1000 μmol m^−2^ s^−1^. The CO_2_ concentration was maintained at 390 ± 5 μmol m^−2^ s^−1^, and the air flux was set at 500 μmol m^−2^ s^−1^. On the 15th, 30th, and 45th day, every second mature leaf was measured 3 times, including the photosynthetic rate (Pn), stomatal conductance (Gs), intercellular CO_2_ concentration (Ci), and transpiration (Tr), for a total of 5 leaves in each treatment. The dark respiration rate (Rr) of the mature leaves was measured at 20:00~22:00 in the dark.

#### 4.3.2. Determination of Photosynthetic Pigment, Soluble Sugar, and Soluble Protein Contents

After 15, 30, and 45 days of treatment, the second mature leaf was used to determine photosynthetic pigment, soluble sugar, and soluble protein contents. In detail, a 0.2 g fresh sample was ground and homogenized with 3 mL of 95% ethanol. Afterwards, 10 mL of the 95% ethanol mixture was added and set for 5 min, until completely white. The filtered extracts were then transferred to a volumetric flask and diluted to 25 mL. An ultraviolet spectrophotometer (UV T5, Shanghai, China) was used to obtain absorbances at 470, 649, and 665 nm.

The Coomassie brilliant blue method was used for the determination of the soluble protein contents. Briefly, 0.2 g sample from each treatment was rapidly weighed. It was then ground into a homogenate and diluted with 10 mL of distilled water. After centrifugation at 4000 rpm/min^−1^ for 10 min at 4°C, 0.1 mL of the supernatant was thoroughly mixed with 5 mL of Coomassie brilliant blue G-250 solution in each tube, allowed to react for 2 min, and then was measured at 595 nm.

The soluble sugars were measured by the anthrone colorimetric method. A total of 0.2 g of fresh sample was weighed and placed in a 10 mL tube, and 10 mL of distilled water was added. After incubation in a water bath for 30 min, the tube was cooled to room temperature. The supernatant (0.5 mL) was mixed with distilled water (1.5 mL), 2% anthrone (0.5 mL), and 98% sulfuric acid (5 mL), and then incubated at 100 °C for 10 min. The absorbance of the sample was read at 630 nm using a UV spectrophotometer (Summit instrument manufacturing Co., Ltd., Shanghai, China).

### 4.4. Analysis of Biochemical Indicators

#### 4.4.1. Determination of Free Amino Acid and Component Contents

Free amino acids were detected according to the National Standard of China (GB/T 8305-2013). Briefly, we added 150 mL of boiling water to 1.0 g of a crushed freeze-dried sample, and then, we extracted it in a 100 °C water bath for 45 min. Ninhydrin colorimetry was adopted to determine the extracts. In addition, the extracts were used to measure the amino acid components followed by GB/T 30987-2020.

#### 4.4.2. Determination of Tea Polyphenol, Catechin, and Caffeine Contents

Tea polyphenols were detected according to the National Standard of China (GB/T 8313-2018). The extraction method was as follows: A total of 0.5 g of crushed freeze-dried sample was placed in a 10 mL centrifuge tube, and then 5 mL of 70% methanol preheated at 70 °C was added. After extraction for 30 min in a 70 °C water bath, the extracted solution was centrifuged to obtain the supernatant. Again, the extraction was repeated with 5 mL of 70% methanol. According to the Folin–Ciocalteu method, tea polyphenols were measured at 765 nm using a UV spectrophotometer (UV T5, Shanghai, China). Meanwhile, the extracts were also prepared to determine the catechin components and caffeine contents using high-performance liquid chromatography (Agilent 1260, Palo Alto, CA, USA). Subsequently, the test parameters were set according to the National Standard.

### 4.5. Analysis of Gene Expressions Related to Theanine Metabolism

At the end of the experiment, new shoots of a three-leaf bud were harvested and ground to extract RNA, according to the manufacture’s protocol (EASYspin Plus Complex Plant RNA Kit, Aidlab Biotechnologies Co., Ltd., Beijing, China). A TRUEscript RT MasterMix (Aidlab Biotechnologies Co., Ltd., Beijing, China) was used to reverse the RNA into cDNA. Primers designed by NCBI (http://www.ncbi.nlm.nih.gov/tools/primer-blast/ (accessed on 18 November 2021)) are listed in Table S7. The qRT-PCR reaction was conducted according to the instructions of 2X M5 HiPer SYBR Premix EsTaq (Mei5 Biotechnology, Co, Ltd., Beijing, China), and the following procedure was set as follows: pre-denaturation at 95 °C for 30 s, denaturation at 95 °C for 5 s, and 40 cycles at 54 °C for 30 s. Three biological replicates were performed in the experiment. Gene expression was calculated using the 2^−ΔΔCt^ method, and *β-actin* was selected as the reference gene.

### 4.6. RNA-Sequencing

For the RNA-sequencing analysis, cDNA libraries were constructed and sequenced by Wuhan Metaware Co., Ltd., Wuhan, China. Total RNA was extracted from the tea leaves using a polysaccharide polyphenol plant total RNA extraction kit (RNAprep Pure). Then, the RNA concentration was evaluated using Qubit 2.0 Flurometer (Life Technologies, Carlsbad, CA, USA), and the quality was assessed using a Bioanalyzer 2100 (Agilent Technologies, Palo Alto, CA, USA), respectively. The cDNA library construction and RNA-sequencing were performed as previously described on an Illumina HiSeq2500 platform [65].

### 4.7. Genome Alignment

After quality control, the clean data were mapped to the tea plant genome using HISAT v2.1.0 software [46,65]. The mapped reads of each sample were assembled using StringTie software, and the unmapped transcripts were defined as new genes.

### 4.8. Differential Expression Analysis and Functional Enrichment Analysis

Fragments per kilobase per million reads (FPKM) were introduced to estimate gene expression levels. DEseq2 v1.22.1 was used to identify differentially expressed genes (DEGs) among different samples, with a threshold of |log_2_fold change| ≥ 1 and FDR < 0.05. Gene function was annotated by NCBI non-redundant protein sequences (Nr), clusters of orthologous groups of proteins (COG/KOG), Swiss-Prot protein sequence database (Swissprot), Kyoto Encyclopedia of Genes and Genomes (KEGG), homologous protein family (Pfam), and Gene Ontology (GO).

### 4.9. The qRT-PCR Validation

Ten DEGs involved in the flavonoid pathway and amino acid metabolism were selected to verify the reliability of the transcriptome data. Primers were also designed by NCBI (http://www.ncbi.nlm.nih.gov/tools/primer-blast/ (accessed on 18 November 2021)) and are listed in Table S8. Quantitative and computational methods were the same as in 4.5.

### 4.10. Statistical Analysis

SPSS 25.0 software (SPSS, Chicago, IL, USA) was used to determine the normality of the data and the homogeneity of the variances. The data from the two tests were subsequently subjected to a one-way analysis of variance (ANOVA) and Duncan’s multiple range tests (*p* < 0.05). OriginPro software (Origin Lab, Berkeley, CA, USA) was used to generate the figures.

## 5. Conclusions

In the present study, the increase in the differences between day and night temperatures hindered the growth of the tea plants, and the net photosynthesis rate and total chlorophyll in the mature leaves decreased. Among the treatments, the tea seedlings treated with a DIF of 10 °C were abundant in caffeine and amino acids, while less abundant in soluble sugars, tea polyphenols, and catechins, which probably resulted from the high expression levels of the genes in the amino acid metabolism, the nitrogen metabolism, as well as the low expression patterns of the genes involved in flavonoid biosynthesis. Overall, the study uncovered the characteristics of growth and metabolism under different DIFs, among which the DIF of 10 °C was most suitable for the growth and development of the tea plants. Our results provided new insights into the differences in the growth and metabolism rates in tea plants under different DIFs, as well as a theoretical basis for optimizing the protected cultivation of tea plants and producing high-quality tea.

## Data Availability

Not applicable.

## Supplementary Materials

The following supporting information can be downloaded at: https://www.mdpi.com/article/10.3390/ijms24076718/s1.
