# Supplementary material for: Characterization of the Difference between Day and Night Temperatures on the Growth, Photosynthesis, and Metabolite Accumulation of Tea Seedlings
Source: Int J Mol Sci. 2023 Apr 4;24(7):6718. doi: 10.3390/ijms24076718 (PMC10095163; doi:10.3390/ijms24076718)
Supplement: Supplementary file 1 [file ijms-24-06718-s001.zip › Supplementary Figure.pdf]

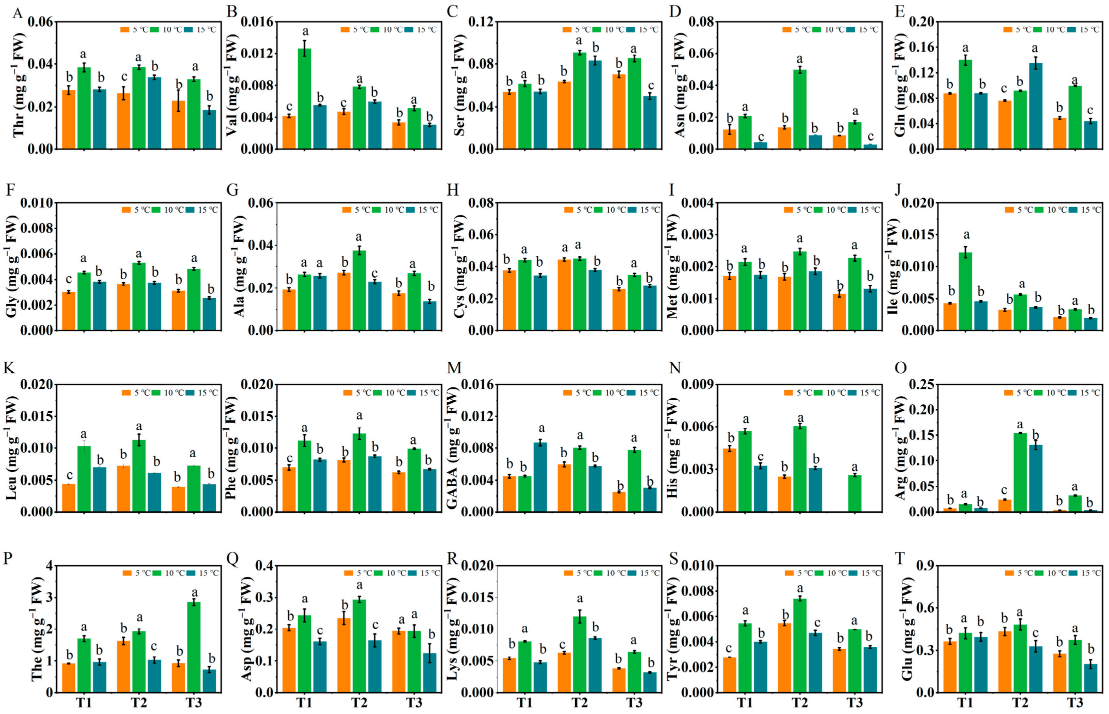

Figure S1. The effects of different DIFs on the contents of amino acid components. (A) Threonine; (B) Valine; (C) Serine; (D) Asparagine; (E) Glutarnine; (F) Glycine; (G) Alanine; (H) Cystine; (I) Methionine; (J) Isoleuine; (K)Leucine; (L) Phenylalanine; (M) γ-aminobutyric acid; (N) Histidine; (O) Arginine; (P) Theanine; (Q) Aspartic acid; (R) Lysine; (S) Tyrosine; (T) Glutamate. Different letters indicate treatments that are significantly different at $p < 0.05$ (n=3).

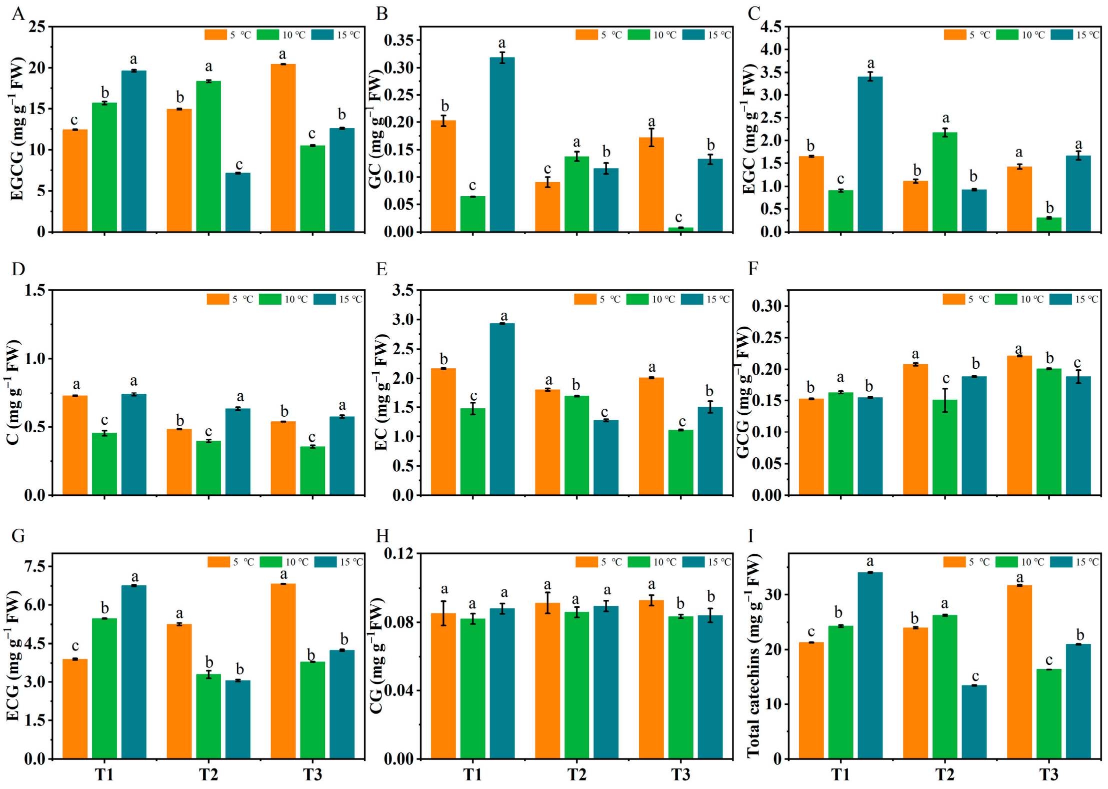

Figure S2. The effects of different DIFs on the contents of catechins components. (A) Epigallocatechin gallate; (B) Gallocatechin; (C) Epigallocatechin; (D) Catechin; (E) Epicatechin; (F) Gallocatechin gallate; (G) Epicatechin gallate; (H) Catechin gallate; (I) Total catechins. Different letters indicate treatments that are significantly different at $p < 0.05$ (n=3).

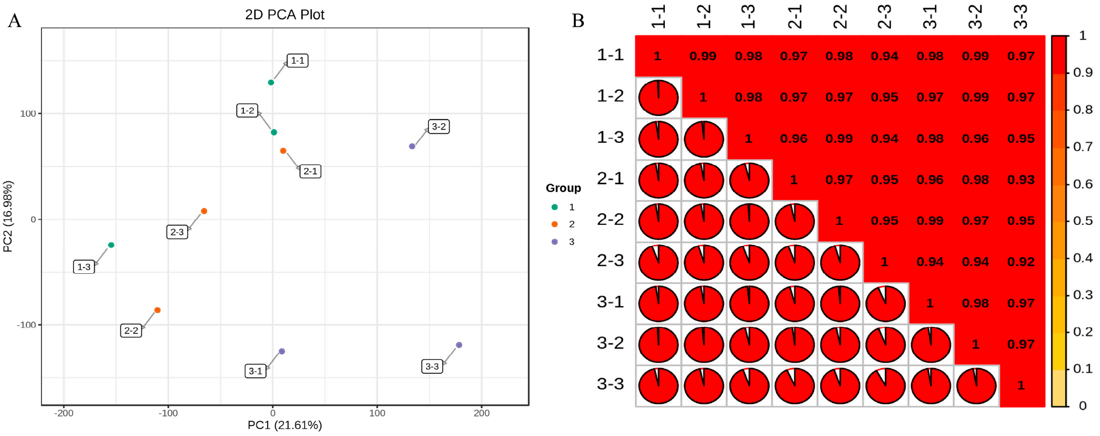

Figure S3. Quality control of transcriptome data. (A) Principal component analysis (PCA); (B) Correlation analysis of samples.

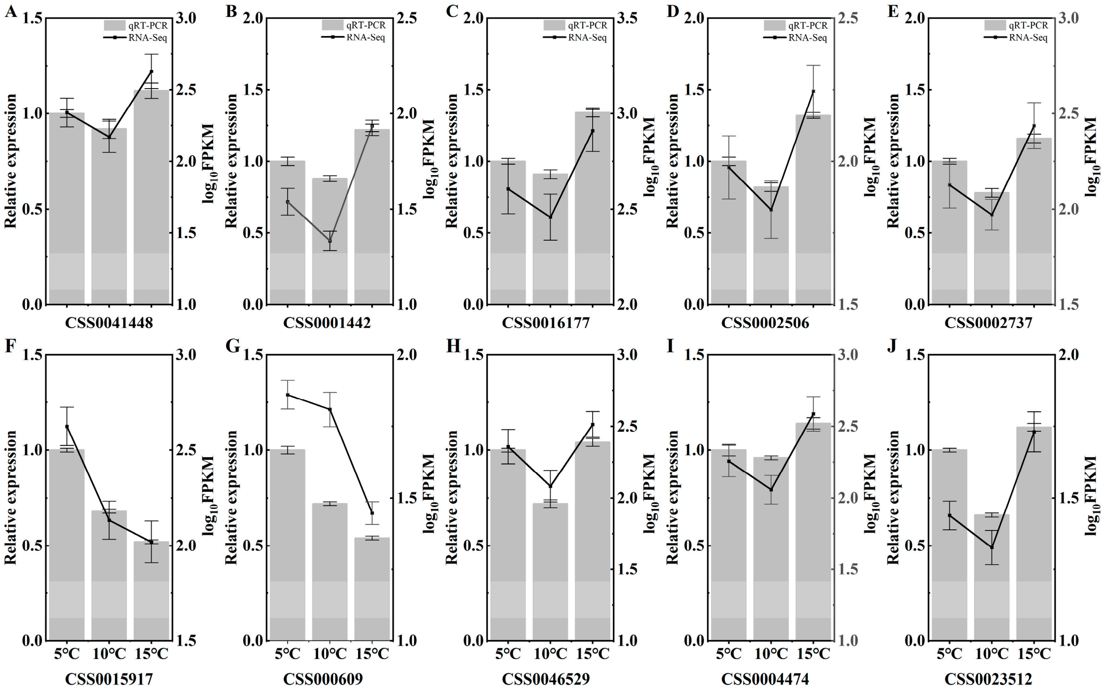

Figure S4. Validation of the differentially expressed genes by qRT-PCR.

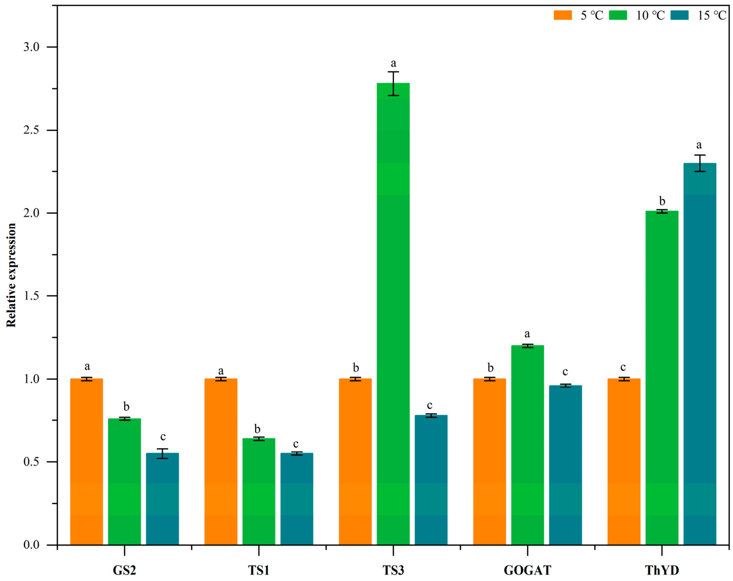

Figure S5. The expression of genes related to the theanine biosynthesis in tea leaves under different DIFs. (A) Glutamine synthetase; (B) Theanine synthetase 1; (C) Theanine synthetase 3; (D) Glutamate synthase; (E) Theanine hydrolase. Different letters indicate treatments that are significantly different at $p < 0.05$ (n=3).
